# Decreasing seroprevalence of herpes simplex virus type 1 and type 2 in Germany leaves many people susceptible to genital infection: time to raise awareness and enhance control

**DOI:** 10.1186/s12879-017-2527-1

**Published:** 2017-07-06

**Authors:** Gerit Korr, Michael Thamm, Irina Czogiel, Christina Poethko-Mueller, Viviane Bremer, Klaus Jansen

**Affiliations:** 1 Postgraduate Training for Applied Epidemiology (PAE, German Field Epidemiology Training Programme), Robert Koch Institute, Berlin, Germany; 2 European Programme for Intervention Epidemiology Training (EPIET), European Centre for Disease Prevention and Control (ECDC), Stockholm, Sweden; 3 Robert Koch Institute (RKI), Berlin, Germany

**Keywords:** Herpes simplex, Herpes genitalis, Health survey, Seroepidemiology, Germany

## Abstract

**Background:**

Herpes simplex infections (HSV1/2) are characterized by recurrent symptoms, a risk of neonatal herpes, and the facilitation of HIV transmission. In Germany, HSV1/2 infections are not notifiable and data are scarce. A previous study found higher HSV1/2 seroprevalences in women in East Germany than in women in West Germany. We assessed changes in the HSV1/2 seroprevalences over time and investigated determinants associated with HSV1/2 seropositivity to guide prevention and control.

**Methods:**

The study was based on the German Health Interview and Examination Survey for Adults (DEGS; 2008–2011) and the German National Health Interview and Examination Survey (GNHIES; 1997–1999). We tested serum samples from DEGS participants for HSV1 and HSV2 immunoglobulin G. We used Pearson’s χ^2^ test to compare the HSV1/HSV2 seroprevalences in terms of sex, age, and region of residence (East/West Germany) and investigated potential determinants by calculating prevalence ratios (PR) with log-binomial regression. All statistical analyses included survey weights.

**Results:**

In total, 6627 DEGS participants were tested for HSV1, and 5013 were also tested for HSV2. Overall, HSV1 seroprevalence decreased significantly from 1997–1999 (82.1%; 95%CI 80.6–83.6) to 2008–2011 (78.4%; 95%CI 77.8–79.7). In the same period, overall HSV2 seroprevalence decreased significantly from 13.3% (95%CI 11.9–14.9) to 9.6% (95%CI 8.6–10.8), notably in 18–24-year-old men (10.4 to 0%) in East Germany. Women were more likely than men to be seropositive for HSV1 (PR 1.1) or HSV2 (PR 1.6). A lower level of education, smoking, and not speaking German were associated with HSV1 in both sexes. Women of older age, who smoked, or had a history of abortion and men of older age or who had not attended a nursery school during childhood were more often seropositive for HSV2.

**Conclusion:**

The reduced seroprevalences of HSV1 and HSV2 leave more people susceptible to genital HSV1/2 infections. Practitioners should be aware of HSV infection as a differential diagnosis for genital ulcers. We recommend educational interventions to raise awareness of the sexual transmission route of HSV1/2, possible consequences, and prevention. Interventions should especially target pregnant women, their partners, and people at risk of HIV.

## Background

Herpes simplex virus is the main cause of genital ulcers worldwide [1]. Both *Herpes simplex virus 1* (HSV1) and *Herpes simplex virus 2* (HSV2) infect the epithelial cells of the skin and mucosa through minor breaks, and then travel by retrograde transport to the sensory root ganglia, where they persist throughout life [2]. Most new infections remain undiagnosed because they are asymptomatic or cause only short-lived symptoms [3]. Clinical lesions typically occur after a primary infection in about 10–25% of infections [4]. Reactivation from the latent state results in the release of the virus from the surface of the skin or mucosa, which is called ‘shedding’ [5]. Viral shedding can occur with or without symptoms and leads to further transmission [4]. The specific tropism of the virus means that HSV1 predominantly infects the orolabial tissue and is transmitted by contact with infected saliva, which often occurs early in life [6]. HSV2 typically infects the genitalia and is transmitted through sexual contact. However, in recent decades, an increasing proportion of genital HSV infections have been caused by HSV1 [7–9]. Two main developments have been suggested to be responsible for this trend: an increased proportion of adolescents and young adults who are HSV1 negative and therefore more susceptible to the acquisition of HSV1 through the sexual route, and an increased frequency of oral sex [10].

The classic clinical presentation of genital herpes infection is characterized by erythematous papules and vesicles on the external genitalia with pain, itching, burning, and, especially in women, dysuria [1]. About 40% of symptomatic men and 70% of symptomatic women present with fever, headache, malaise and myalgias. Complications include aseptic meningitis, extragenital lesions and autonomic dysfunction including urinary detention. Genital herpes can be associated with psychosocial consequences including anger, low self-esteem, fear of rejection by sexual partners, and depression.

Approximately 57 and 89% of individuals with a history of primary HSV1 or HSV2 infection, respectively, experience symptomatic HSV reactivation (recurrence) with symptoms lasting between 5 and 10 days [1, 11]. Individuals with genital HSV2 infection experience about four recurrences per year whereas those with genital HSV1 infection experience about one recurrence per year [11]. Prospective follow-up has shown a reduction of recurrences over time in most but not all patients [1].

Genital herpes infection can be transmitted from mother to child, with primary infection in a mother close to delivery or within the last trimester being the greatest risk [4]. Symptoms in the neonate include skin and eye disease, encephalitis, or disseminated infection [12]. Cognitive impairment, severe neurological disease, organ dysfunction, and death are among the common sequelae [1]. It has also been shown that genital herpes increases the risk of acquiring *Human immunodeficiency virus* (HIV), HIV transmission, and HIV progression [13, 14].

In 2012, an estimated 3709 million people aged 0–49 years were infected with HSV1 worldwide and an estimated 417 million people aged 15–49 years were living with HSV2, which constitutes a global HSV1 seroprevalence of 67% and a global HSV2 seroprevalence of 11.3% [15, 16].

In the World Health Organization European region, approximately 207 million women and 187 million men aged 0–49 years were living with HSV1 in 2012, corresponding to seroprevalences of 69 and 61%, respectively [15]. In the same region, 21.7 million women and 9.7 million men aged 15–49 years were estimated to be HSV2 seropositive [16]. In national cross-sectional serological surveys performed in European countries between 1989 and 2000, the age-standardized HSV1 seroprevalence ranged from 52% in Finland to 84% in Bulgaria and the age-standardized HSV2 seroprevalence ranged from 4% in England and Wales to 24% in Bulgaria [17].

A German survey based on representative data collected in 1997–1999 found an overall age-standardized HSV1 seroprevalence of 82.6% and an overall HSV2 seroprevalence of 13.3% [18]. Interestingly, women residing in the area of the former German Democratic Republic (GDR), now the east of unified Germany, had significantly higher age-adjusted seroprevalences of HSV1 and HSV2 than women residing in the former Federal Republic of Germany (FRG), now the west of unified Germany. The authors of that study discussed a potentially different sexual behavior of these women as a possible explanation for these disparities [18].

The different political and economic system of the GDR which ceased to exist in 1990 impacted the East German society profoundly. The contrast between the GDR and the FRG included differences in family planning, partner relationships and sexual behavior [19]. For example, in the GDR, sexual health education at schools was not very common, hormonal contraception was available free of charge, and use of condoms was less common when compared to the FRG. After reunification, risk perception and sexual behavior of East Germans started to change gradually. From 1990 to 2000 differences between the incidence of syphilis (median incidence GDR vs. FRG: 2.1 vs. 1.3 per 100.000 population) and gonorrhea (median incidence GDR vs. FRG: 7.2 vs. 4.1 per 100.000 population) decreased from 3.5- to 1.7-fold for syphilis and from 2.1- to 1.1-fold for gonorrhea [18]. Whereas in 1994 the number of 14–17-year-olds which affirmed to have sexual health education at schools differed between former GDR (around 45%) and former FRG (around 83%), in 2014 about 95% of 14–17-year-olds affirmed to have sexual health education at their schools, regardless whether they lived in the former GDR or former FRG [20].

Given the described high seroprevalence of HSV1 and HSV2 in Germany [18] and the recurrent nature of the infections, the clinical and psychosocial burden of the genital ulcer disease caused by HSV is probably very high. However, there has been little research into genital herpes in Germany, possibly because it is not reportable in Germany and there are no specialized sexual health clinics with an established routine surveillance strategy for sexually transmitted infections (STIs).

In this paper, we provide recent representative data and describe the latest developments in HSV1 and HSV2 seroepidemiology in Germany to guide prevention and control of HSV1/2 infections and to promote research into this important public-health issue. Based on a large nationwide representative survey undertaken in 2008–2011, we assessed the seroprevalence of HSV1 and HSV2 in adults in Germany, compared these seroprevalences to the findings of a previous survey in 1997–1999, and investigated factors such as sociodemographic variables and sexual behavior associated with HSV1 and HSV2 seropositivity.

## Methods

### Study design and population

The study was based on the German National Health Interview and Examination Survey (GNHIES) and the German Health Interview and Examination Survey for Adults (DEGS), which were conducted in 1997–1999 and 2008–2011, respectively [21, 22]. Both studies are part of the national health monitoring conducted by the Robert Koch Institute (RKI).

The GNHIES was based on a stratified, multistage, cross-sectional, national representative sample of individuals aged 18–79 years from the noninstitutionalized population of Germany. It had a total of 7124 participants corresponding to a 61.5% response proportion (18).

Nationwide representative data on the health status of the adult (18–79 years) German resident population were collected in the DEGS. In total, 8151 individuals participated. The response proportion was 48.4%. The analysis of nonresponder questionnaires revealed high population representativeness. The survey design was both cross-sectional and longitudinal. Almost half of all the participants were already enrolled in the GNHIES.

Both surveys included questionnaires, physical tests, and the collection of biomaterial. The sampling design and data collection of both surveys are described in detail elsewhere [21, 22]. The DEGS questionnaire included variables involving self-reported morbidity, medication use, symptoms and complaints, mental health, subjective health, sex-specific health issues, injuries, falls, functional capacities, disability, health-related behavior, living and social conditions, sociodemographic context variables, and health-care services utilization. In our study, we used the following DEGS variables: sex, age, educational classification, income, employment status, current smoking, number of other children in the household during childhood, attendance at a nursery school during childhood, degree of urbanization, region of residence, German mother tongue, number of sexual partners in the preceding 12 months, HSV1/2 serostatus, current use of birth control methods (only women were asked), miscarriage, abortion, and condom use (women were only asked about condom use in the context of birth control methods, so this variable could only be used for men in terms of general safer sex behavior).

### Laboratory methods

Serum samples from the DEGS participants were tested for HSV1 (gG1) and HSV2 (gG2) with a chemiluminescence immunoassay (CLIA) (LIAISON® HSV1/2, DiaSorin, von Hevesy-Strasse 3, 63,128 Dietzenbach). The light signal, and hence the amount of isoluminol-antibody conjugate, was measured by a photomultiplier as relative light units (RLU). An index value of <0.9 was defined as immunoglobulin G (IgG) negative and an index value >1.1 as IgG positive. Because retesting samples with an equivocal result for HSV using immunoblotting usually produces a negative result and equivocal results have been classified as negative in other seroprevalence studies [17], we classified all of our equivocal samples (0.3% of all the samples for HSV1 and 0.8% of all the samples for HSV2) as negative.

In a previous seroepidemiological survey of HSV1/2, researchers tested serum samples from 3792 GNHIES participants with another indirect ELISA (MRL Diagnostics, Los Angeles, CA, USA, now HerpesSelect®, Focus Technologies, Cypress, CA, USA) [18].

It has been demonstrated that both assays compare well in terms of their sensitivity and specificity, with almost 100% concordance in comparative analyses [23, 24].

### Statistical analyses

All statistical analyses included survey weights based on sex, age, federal state of residence, municipality size, nationality (German yes/no), and education level to account for any deviations of the survey sample from German population statistics [21].

Using the more recent survey (DEGS), we:

- calculated the overall and age- and sex-specific HSV1 and HSV2 seroprevalences and their 95% confidence intervals (CIs);
- used Pearson’s χ^2^ test to investigate the potential associations between seroprevalence and sex, age, and region of residence (East or West Germany); and
- investigated potential determinants associated with HSV1/2 seropositivity by calculating the PRs using log-binomial regression. Because the questions concerning sexual behavior differed for men and women, we performed the univariable and multivariable analyses separately for each sex. If a *p*-value was <0.2 in the univariable analysis, we included that variable in a stepwise forward variable selection to find a suitable multivariable model. As only a small number of behavioral variables were available within the survey, we set the cutoff for the *p*-value at 0.2 to take these variables sufficiently into account. Two-way interaction terms were generated for biologically relevant covariate pairs, and retained in the model if significant (*p* ≤ 0.05).

To examine changes in the HSV1 and HSV2 seroprevalences between 1997–1999 and 2008–2011 in 18–64-year-olds in Germany, we also used the HSV1 and HSV2 test results from GNHIES and applied Pearson’s χ^2^ test. These analyses were adjusted for the reparticipation rate of the GNHIES participants in the DEGS, in addition to the above mentioned weighting procedure [21].

All data analyses were performed with Stata 14 (*Stata Statistical Software: Release 14*. StataCorp LP, College Station, TX).

## Results

### Characteristics of the study participants

A total of 6627 adults, representing 91.6% of the DEGS survey population with available blood samples, were tested for HSV1 IgG. Of these, 5013 were also tested for HSV2 IgG. HSV1/2 were not tested in the other participants because the amount of blood available for all the tests performed in the survey was limited. Neither the study population tested for HSV1 nor the study population tested for HSV2 differed from the total DEGS study population with regard to sex, age, or region of residence.

### Weighted seroprevalence of HSV1

**Fig. 1:**
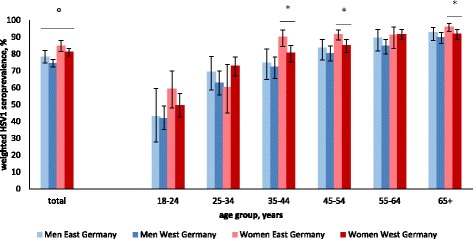
Weighted HSV1 seroprevalence according to sex, age and region of residence, Germany 2008–2011. *HSV1 seroprevalence differed significantly between women and men. °HSV1 seroprevalence differed significantly between women in East Germany vs. women in West Germany in the age groups 35–44, 45–54 and 65+

The overall seroprevalence of HSV1 in the DEGS was 78.7% (95%CI 77.2–80.1). The HSV1 seroprevalence was significantly higher in women (82.0%, 95%CI 80.0–83.7) than in men (75.4%, 95%CI 73.4–77.3). HSV1 seropositivity increased with age from 46.8% (95%CI 42.4–51.2) in the 18–24-year age group to 91.9% (95%CI 89.8–93.6) in the 65+ −year age group. HSV1 seroprevalence was significantly higher in residents of East Germany (81.7%, 95%CI 79.0–84.1) than in residents of West Germany (77.9%, 95%CI 76.2–79.5). However, when stratified by sex, this effect was only significant for women in the age groups 35–44 years (East: 90.2%, 95%CI 84.3–94.1; West: 80.7, 95%CI 75.3–85.1), 45–54 years (East: 91.7%, 95%CI 88.3–94.1; West: 85.1%, 95%CI 80.9–88.5), and 65+ years (East: 96.2%, 95%CI 93.3–97.9; West: 92.1, 95%CI 88.6–94.5) (Fig. 1). In women aged 25–34 and 55–64 years, the effect was reversed: the women in West Germany had a higher HSV1 seroprevalence than their counterparts in East Germany, although the difference was not statistically significant.

### Weighted seroprevalence of HSV2

**Fig. 2:**
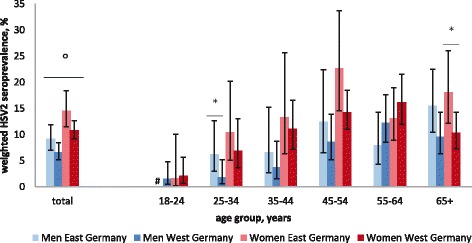
Weighted HSV2 seroprevalence according to sex, age and region of residence, Germany 2008–2011. *HSV2 seroprevalence differed significantly between women and men. °HSV2 seroprevalence differed significantly between 25-34 year old men in East vs. West Germany and in women aged 65+ in East vs. West Germany. ^#^There were no HSV2 positive results among 18-24 year old male study participants living in East Germany

The overall seroprevalence of HSV2 in the DEGS was 9.4% (95%CI 8.3–10.5). HSV2 seroprevalence was significantly higher in women (11.7%, 95%CI 10.2–13.3) than in men (7.2%, 95%CI 5.9–8.6). HSV2 seropositivity increased from 1.6% (95%CI 0.8–3.2) in the 18–24-year age group to 13.4% (95%CI 10.9–16.4) in the 55–64-year age group, decreasing thereafter to 11.7% (95%CI 9.6–14.2) in study participants aged 65+. The seroprevalence of HSV2 was significantly higher in the residents of East Germany (11.9%, 95%CI 9.9–14.3) than in the residents of West Germany (8.7%, 95%CI 7.5–10.0). However, when stratified by sex, only women in East Germany had a higher seroprevalence than their counterparts in West Germany (East: 14.6%, 95%CI 11.5–18.4; West: 10.8%, 95%CI 9.2–12.7). When stratified by age and sex, the higher HSV2 seroprevalence in East Germany was only significant for women aged 65+ years (East: 18.1%, 95%CI 12.2–26.0; West: 10.3%, 95%CI 7.3–14.3) and men aged 25–34 years (East: 6.2%, 95%CI 3.0–12.6; West: 1.8%; 95%CI 0.6–5.2) (Fig. 2). The opposite trend was observed in both women and men aged 18–24 and 55–64 years, although the differences were not statistically significant.

### Factors associated with HSV1 seropositivity

In the DEGS, women were more likely to be HSV1 IgG positive than men (crude prevalence ratio [CPR] 1.1; 95%CI 1.1–1.1). The results of the univariable analyses, stratified by sex, are shown in Tables 1 and 2.

**Table 1:** Associations between variables and HSV1 seropositivity in women, Germany 2008–2011

Variables	N	Weighted^a^ prevalence %	(95% CI)	Univariable Analysis		Multivariable Analysis	
				PR^b^(95% CI)	*p*-value	PR^c^(95% CI)	*p*-value
Age group (years)							
18–24	283	51.4	(45.3–57.5)	Ref	Ref	Ref	Ref
25–34	400	70.4	(64.6–75.5)	*1.4 (1.2–1.6)*	*0.000*	1.2 (1.0–1.4)	0.057
35–44	519	82.4	(77.9–86.1)	*1.6 (1.4–1.8)*	*0.000*	*1.4 (1.2–1.6)*	*0.000*
45–54	714	86.5	(83.1–89.2)	*1.7 (1.5–1.9)*	*0.000*	*1.4 (1.2–1.7)*	*0.000*
55–64	630	91.8	(88.7–94.1)	*1.8 (1.6–2.0)*	*0.000*	*1.5 (1.3–1.8)*	*0.000*
65+	895	93.1	(90.4–95.1)	*1.8 (1.6–2.0)*	*0.000*	*1.6 (1.3–1.8)*	*0.000*
CASMIN educational classification							
Low	1117	90.0	(87.6–91.9)	*1.3 (1.2–1.4)*	*0.000*	*1.2 (1.1–1.3)*	*0.000*
Medium	1772	79.3	(76.4–82.0)	*1.2 (1.1–1.2)*	*0.000*	*1.2 (1.1–1.3)*	*0.000*
High	531	68.8	(63.7–73.5)	Ref	Ref	Ref	Ref
Income							
Low	991	83.4	(80.1–86.2)	Ref	Ref	−	−
Medium	2031	82.0	(79.7–84.2)	1.0 (0.9–1.0)	0.481	−	−
High	419	76.9	(70.6–82.2)	0.9 (0.8-1.0)	0.066	−	−
Employment situation							
Worker	827	91.2	(88.5–93.4)	*1.1 (1.1–1.2)*	*0.000*	*1.1 (1.0–1.1)*	*0.004*
Employee	1611	81.4	(78.5–83.9)	Ref	Ref	Ref	Ref
Civil servant	169	70.3	(60.5–78.5)	*0.9 (0.8–1.0)*	*0.036*	0.9 (0.8–1.1)	0.482
Free-lance/self-reliant	233	84.9	(77.9–90.0)	1.0 (1.0–1.1)	0.232	1.0 (1.0–1.1)	0.379
Family worker	64	87.5	(75.2–94.1)	1.1 (1.0–1.2)	0.201	1.0 (0.9–1.2)	0.551
other	124	51.8	(40.9–62.5)	*0.6 (0.5–0.8)*	*0.000*	0.8 (0.6–1.0)	0.086
Smoking, currently							
Daily	643	84.2	(80.0–87.7)	1.0 (1.0–1.1)	0.266	*1.1 (1.0–1.1)*	*0.035*
Occasionally	179	75.1	(67.2–81.6)	0.9 (0.8-1.0)	0.090	*1.1 (1.0–1.2)*	*0.023*
No, not anymore	825	82.1	(77.7–85.7)	1.0 (1.0–1.1)	0.841	1.0 (0.9–1.1)	0.898
Never smoked	1776	81.6	(79.1–83.9)	Ref	Ref	Ref	Ref
Number of other children in household during childhood							
0	369	76.6	(70.6–81.7)	Ref	Ref	−	−
1–2	1478	74.9	(71.9–77.7)	1.0 (0.9–1.1)	0.579	−	−
> 2	1594	90.6	(88.5–92.3)	*1.2 (1.1–1.3)*	*0.000*	−	−
Visit of nursery during childhood							
No	801	87.9	(84.4–90.7)	*1.2 (1.1–1.2)*	*0.000*	−	−
Yes	1705	74.8	(71.9–77.5)	Ref	Ref	−	−
Degree of urbanization							
Rural	607	83.8	(78.3–88.2)	Ref	Ref	−	−
Provincial	844	84.0	(80.1–87.3)	1.0 (0.9–1.1)	0.956	−	−
Urban	963	80.2	(76.3–83.6)	1.0 (0.8–1.0)	0.238	−	−
Metropolitan	1027	81.1	(77.7–84.0)	1.0 (0.9–1.0)	0.347	−	−
Region of residence							
West Germany	2350	81.2	(78.9–83.2)	Ref	Ref	−	−
East Germany (including Berlin)	1091	85.0	(81.4–88.0)	1.0 (1.0-1.1)	0.052	−	−
German mother tongue							
Yes	3170	81.0	(78.9–83.0)	Ref	Ref	Ref	Ref
No	242	88.4	(82.2–92.6)	*1.1 (1.0–1.2)*	*0.000*	*1.1 (1.1–1.2)*	*0.000*
Number of sexual partners in last 12 months							
None	1051	85.4	(82.3–88.0)	*1.0 (1.0–1.1)*	*0.043*	−	−
1	2110	81.5	(78.9–83.9)	Ref	Ref	−	−
2–3	153	62.3	(53.1–70.7)	*0.8 (0.7–0.9)*	*0.001*	−	−
> 3	24	76.6	(49.1–91.8)	0.9 (0.7–1.2)	0.668	−	−
HSV2 serostatus							
HSV2 seronegative	2262	81.6	(79.1–83.8)	Ref	Ref	−	−
HSV2 seropositive	323	88.9	(83.7–92.6)	*1.1 (1.0–1.2)*	*0.004*	−	−
Current use of birth control methods							
Yes	978	72.0	(68.5–75.3)	Ref	Ref	−	−
No	1523	84.1	(81.6–86.3)	*1.2 (1.1–1.2)*	*0.000*	−	−
Which birth control method							
-Contraceptive pill							
Yes	470	67.2	(62.1–72.0)	Ref	Ref	−	−
No	478	77.1	(72.1–81.5)	*1.1 (1.0–1.3)*	*0.005*	−	−
-Condoms							
Yes	287	71.8	(65.3–77.6)	Ref	Ref	−	−
No	661	72.3	(67.9–76.4)	1.0 (0.9–1.1)	0.891	−	−
Miscarriage							
None	2008	77.1	(74.5–79.5)	Ref	Ref	−	−
1 or more	549	88.3	(83.8–91.6)	*1.1 (1.1–1.2)*	*0.000*	−	−
Abortion							
None	2047	77.3	(74.8–79.7)	Ref	Ref	−	−
1 or more	471	88.5	(84.0–91.9)	*1.1 (1.1–1.2)*	*0.000*	−	−

In italics: PR is statistically significant (*p* < 0.05)

^a^We used survey weights to account for deviations of the survey sample from the sampling parameters (i.e. age, sex, region, urban/rural region, community size, citizenship and education)

^b^If a *p*-value was <0.2 in the univariable analysis, we included that variable in a stepwise forward variable selection to find a suitable multivariable model

^c^Adjusted prevalence rates (PR) of variables which stayed in the final model (*p*-value ≤0.05) are reported

**Table 2:** Associations between variables and HSV1 seropositivity in men, Germany 2008–2011

Variables	N	Weighted^a^ prevalence %	(95% CI)	Univariable Analysis		Multivariable Analysis	
				PR^b^ (95% CI)	*p*-value	PR^c^ (95% CI)	*p*-value
Age group (years)							
18–24	289	42.2	(35.8–48.9)	Ref	Ref	Ref	Ref
25–34	364	64.5	(58.4–70.2)	*1.5 (1.3–1.8)*	*0.000*	*1.5 (1.2–1.8)*	*0.000*
35–44	448	72.8	(67.1–77.9)	*1.7 (1.5–2.0)*	*0.000*	*1.7 (1.4–2.0)*	*0.000*
45–54	627	81.2	(77.1–84.6)	*1.9 (1.6–2.3)*	*0.000*	*1.9 (1.6–2.2)*	*0.000*
55–64	559	85.9	(81.8–89.2)	*2.0 (1.7–2.4)*	*0.000*	*2.0 (1.7–2.3)*	*0.000*
65+	899	90.6	(87.5–92.9)	*2.1 (1.8–2.5)*	*0.000*	*1.9 (1.6–2.3)*	*0.000*
CASMIN educational classification							
Low	1045	85.2	(82.2–87.7)	*1.2 (1.1–1.3)*	*0.000*	*1.2 (1.1–1.2)*	*0.000*
Medium	1407	69.7	(66.4–72.8)	1.0 (0.9–1.1)	0.967	1.1 (1.0–1.2)	0.062
High	714	69.8	(65.0–74.2)	Ref	Ref	Ref	Ref
Income							
Low	855	79.1	(75.6–82.2)	Ref	Ref	−	−
Medium	1837	74.2	(71.6–76.6)	*0.9 (0.9–1.0)*	*0.015*	−	−
High	494	72.3	(67.1–76.9)	*0.9 (0.8–1.0)*	*0.022*	−	−
Employment situation							
Worker	1109	80.2	(77.0–83.0)	*1.1 (1.0–1.2)*	*0.004*	−	−
Employee	1033	73.2	(69.4–76.8)	Ref	Ref	−	−
Civil servant	224	76.2	(68.3–82.6)	1.0 (0.9–1.2)	0.462	−	−
Free-lance/self-reliant	394	78.6	(72.0–84.0)	*1.1 (1.0-1.2)*	*0.133*	−	−
Family worker	6	100.0		*1.4 (1.3–1.4)*	*0.002*	−	−
Other	93	48.0	(35.7–60.5)	*0.7 (0.5–0.9)*	*0.000*	−	−
Smoking, currently							
Daily	706	76.1	(72.0–79.8)	*1.1 (1.0–1.2)*	*0.040*	1.1 (1.0–1.1)	0.060
Occasionally	201	74.0	(65.4–81.1)	1.0 (0.9–1.2)	0.440	*1.1 (1.0–1.3)*	*0.013*
No, not anymore	1181	79.5	(76.2–82.4)	*1.1 (1.1–1.2)*	*0.000*	1.0 (0.9–1.1)	0.985
Never smoked	1081	70.6	(66.7–74.2)	Ref	Ref	Ref	Ref
Number of other children in household during childhood							
0	341	70.0	(63.5–75.7)	Ref	Ref	Ref	Ref
1–2	1323	64.8	(61.7–67.7)	0.9 (0.8-1.0)	0.127	0.9 (0.9–1.0)	0.184
> 2	1522	88.1	(86.0–90.0)	*1.3 (1.1–1.4)*	*0.000*	*1.1 (1.0–1.2)*	*0.039*
Visit of nursery during childhood							
No	726	80.5	(76.8–83.7)	Ref	Ref	−	−
Yes	152	67.1	(64.1–70.0)	*1.2 (1.1–1.3)*	*0.000*	−	−
Degree of urbanization							
Rural	636	77.2	(73.2–80.7)	Ref	Ref	−	−
Provincial	799	77.3	(73.3–80.8)	1.0 (0.9–1.1)	0.974	−	−
Urban	866	72.8	(68.8–76.4)	0.9 (0.9-1.0)	0.105	−	−
Metropolitan	885	75.3	(71.6–78.8)	1.0 (0.9–1.0)	0.490	−	−
Region of residence							
West Germany	2151	74.6	(72.3–76.7)	Ref	Ref	Ref	Ref
East Germany (including Berlin)	1035	78.4	(74.5–81.9)	1.1 (1.0-1.1)	0.075	*1.1 (1.0–1.1)*	*0.003*
German mother tongue							
Yes	2952	73.8	(71.7–75.8)	Ref	Ref	Ref	Ref
No	208	86.7	(79.6–91.6)	*1.2 (1.1–1.3)*	*0.000*	*1.2 (1.1–1.3)*	*0.000*
Number of sexual partners in last 12 months							
None	632	74.1	(68.6–78.9)	1.0 (0.9–1.0)	0.414	−	−
1	2113	76.4	(74.0–78.6)	Ref	Ref	−	−
2–3	205	65.2	(57.1–72.5)	*0.9 (0.7–1.0)*	*0.012*	−	−
> 3	98	68.8	(57.0–78.6)	0.9 (0.8-1.1)	0.193	−	−
HSV2 serostatus							
HSV2 seronegative	2221	74.8	(72.5–76.9)	Ref	Ref	−	−
HSV2 seropositive	207	77.4	(69.6–83.6)	1.0 (0.9–1.1)	0.472	−	−
Use of condoms							
Generally	398	59.3	(53.7–64.7)	Ref	Ref	−	−
Occasionally	400	67.5	(61.6–73.0)	*1.1 (1.0-1.3)*	*0.034*	−	−
No	1881	78.0	(77.3–82.4)	*1.3 (1.2–1.5)*	*0.000*	−	−
N/a	372	79.1	(72.6–84.3)	*1.3 (1.2–1.5)*	*0.000*	−	−

In italics: PR is statistically significant (*p* < 0.05)

^a^We used survey weights to account for deviations of the survey sample from the sampling parameters (i.e. age, sex, region, urban/rural region, community size, citizenship and education)

^b^If a *p*-value was <0.2 in the univariable analysis, we included that variable in a stepwise forward variable selection to find a suitable multivariable model

^c^Adjusted prevalence rates (PR) of variables which stayed in the final model (*p*-value ≤0.05) are reported

Among women, older age, a lower level of education, being employed as a ‘worker’ rather than an ‘employee’, smoking, and not speaking German as a first language were associated with HSV1 seropositivity in the multivariable model (Table 1).

Among men, older age, a lower level of education, smoking, more than two other children in the household during childhood, residence in East Germany, and not speaking German as a first language were associated with HSV1 seropositivity in the multivariable model (Table 2). We found no significant interactions among the relevant covariate pairs for either men or women.

### Factors associated with HSV2 seropositivity

Women were also more likely to be seropositive for HSV2 than men (CPR 1.6; 95%CI 1.3–2.0). The results of the univariable analysis, stratified by sex, are shown in Tables 3 and 4. We found that older age, smoking, and a history of abortion were associated with HSV2 seropositivity in women (Table 3) and that older age, not attending a nursery school during childhood, and the occasional use of condoms (as opposed to their consistent use) were associated with HSV2 seropositivity in men (Table 4). We found no relevant covariate pairs.

**Table 3:** Associations between variables and HSV2 seropositivity in women, Germany 2008–2011

Variables	N	Weighted^a^ prevalence %	(95% CI)	Univariable Analysis		Multivariable Analysis	
				PR^b^ (95% CI)	*p*-value	PR^c^ (95% CI)	*p*-value
Age group (years)							
18–24	227	2.0	(0.8–4.8)	Ref	Ref	Ref	Ref
25–34	277	7.6	(4.5–12.5)	*3.9 (1.4–11.1)*	*0.012*	*3.5 (1.2–10.4)*	*0.021*
35–44	405	11.5	(7.9–16.4)	*5.9 (2.3–15.2)*	*0.000*	*5.6 (2.1–14.7)*	*0.001*
45–54	535	16.1	(12.8–20.0)	*8.2 (3.2–21.2)*	*0.000*	*7.3 (2.7–19.5)*	*0.000*
55–64	461	15.5	(12.0–19.8)	*7.9 (3.3–18.9)*	*0.000*	*8.0 (3.1–20.5)*	*0.000*
65+	680	12.3	(9.4–15.6)	*6.3 (2.4–16.3)*	*0.000*	*6.5 (2.5–17.0)*	*0.000*
CASMIN educational classification							
Low	841	10.5	(8.2–13.3)	1.2 (0.7–1.8)	0.522	−	−
Medium	1335	12.6	(10.6–15.0)	1.4 (0.9-2.1)	0.131	−	−
High	392	9.1	(6.3–13.0)	Ref	Ref	−	−
Income							
Low	750	10.8	(8.2–14.1)	Ref	Ref	−	−
Medium	1529	11.7	(9.8–13.8)	1.1 (0.8–1.5)	0.652	−	−
High	306	14.5	(10.2–20.2)	1.3 (0.9–2.1)	0.204	−	−
Employment situation							
Worker	623	12.4	(9.6–16.0)	1.0 (0.7–1.3)	0.884	−	−
Employee	1174	12.7	(10.5–15.4)	Ref	Ref	−	−
Civil servant	128	14.5	(8.6–23.4)	1.1 (0.7–1.9)	0.623	−	−
Free-lance/self-reliant	174	13.2	(8.3–20.5)	1.0 (0.6–1.7)	0.874	−	−
Family worker	47	5.3	(1.4–18.8)	0.4 (0.1–1.6)	0.202	−	−
Other	96	4.0	(1.4–10.8)	*0.3 (0.1–0.9)*	*0.033*	−	−
Smoking, currently							
Daily	486	15.2	(11.8–19.2)	*1.5 (1.1–2.1)*	*0.006*	*1.6 (1.0–2.4)*	*0.033*
Occasionally	133	8.7	(4.9–15.1)	0.9 (0.5–1.6)	0.673	1.1 (0.5–2.3)	0.847
No, not anymore	607	13.4	(10.6–16.5)	*1.3 (1.0–1.8)*	*0.031*	1.2 (0.9–1.7)	0.226
Never smoked	1345	9.9	(8.1–12.0)	Ref	Ref	Ref	Ref
Number of other children in household during childhood							
0	272	12.5	(8.2–18.6)	Ref	Ref	−	−
1–2	1125	9.6	(8.0–11.6)	0.8 (0.5–1.2)	0.221	−	−
> 2	1188	13.6	(11.1–16.5)	1.1 (0.7–1.7)	0.728	−	−
Visit of nursery during childhood							
No	614	14.5	(11.7–17.8)	*1.5 (1.1–2.0)*	*0.008*	−	−
Yes	1264	9.7	(7.8–12.0)	Ref	Ref	−	−
HSV1 serostatus							
HSV1 seronegative	431	7.3	(4.8–11.1)	Ref	Ref	−	−
HSV1 seropositive	2154	12.6	(10.9–14.5)	*1.7 (1.1–2.7)*	*0.017*	−	−
Degree of urbanization							
Rural	424	9.6	(6.2–14.6)	Ref	Ref	−	−
Provincial	651	11.3	(8.5–14.7)	1.2 (0.7–2.0)	0.539	−	−
Urban	792	10.1	(8.0–12.7)	1.1 (0.6–1.7)	0.835	−	−
Metropolitan	718	14.4	(11.7–17.7)	1.5 (0.9-2.4)	0.095	−	−
Region of residence							
West Germany	1778	10.8	(9.2–12.7)	Ref	Ref	−	−
East Germany (including Berlin)	807	14.6	(11.5–18.4)	*1.3 (1.0–1.8)*	*0.037*	−	−
German mother tongue							
Yes	2390	11.5	(9.9–13.3)	Ref	Ref	−	−
No	172	10.9	(6.9–16.8)	0.9 (0.8–1.5)	0.825	−	−
Number of sexual partners in last 12 months							
None	788	12.7	(10.2–15.7)	1.1 (0.8–1.5)	0.500	−	−
1	1588	11.5	(9.6–13.7)	Ref	Ref	−	−
2–3	116	12.0	(5.3–24.8)	1.0 (0.5–2.3)	0.917	−	−
> 3	17	10.3	(1.4–49.0)	0.9 (0.1–6.2)	0.912	−	−
Current use of birth control methods^d^							
Yes	740	8.5	(6.3–11.3)	Ref	Ref	−	−
No	1140	14.1	(12.0–16.6)	*1.7 (1.2–2.3)*	*0.003*	−	−
Which birth control method							
-Contraceptive Pill							
Yes	363	7.0	(4.4–10.8)	Ref	Ref	−	−
No	353	9.6	(6.5–14.0)	1.4 (0.8–2.4)	0.264	−	−
-Condoms							
Yes	217	8.0	(4.7–13.4)	Ref	Ref	−	−
No	499	8.4	(5.7–12.3)	1.0 (0.5–2.1)	0.892	−	−
Miscarriage							
None	1504	10.4	(8.5–12.6)	Ref	Ref	−	−
1 or more	424	15.9	(12.1–20.6)	*1.5 (1.1–2.1)*	*0.009*	−	−
Abortion							
None	1538	10.1	(8.4–12.2)	Ref	Ref	Ref	Ref
1 or more	351	20.1	(15.3–25.9)	*2.0 (1.4–2.7)*	*0.000*	*1.5 (1.1–2.2)*	*0.023*

In italics: PR is statistically significant (*p* < 0.05)

^a^We used survey weights to account for deviations of the survey sample from the sampling parameters (i.e. age, sex, region, urban/rural region, community size, citizenship and education)

^b^If a *p*-value was <0.2 in the univariable analysis, we included that variable in a stepwise forward variable selection to find a suitable multivariable model

^c^Adjusted prevalence rates (PR) of variables which stayed in the final model (*p*-value ≤0.05) are reported

^d^Women older than 65 years of age were not asked about their current use of birth control methods

**Table 4:** Associations between variables and HSV2 seropositivity in men, Germany 2008–2011

Variables	N	Weighted^a^ prevalence %	(95% CI)	Univariable Analysis		Multivariable Analysis	
				PR^b^ (95% CI)	*p*-value	PR^c^ (95% CI)	*p*-value
Age group (years)							
18–24	222	1.2	(0.4–3.9)	Ref	Ref	Ref	Ref
25–34	283	2.8	(1.4–5.3)	2.3 (0.6–9.0)	0.247	2.0 (0.5–7.8)	0.314
35–44	343	4.3	(2.2–8.2)	3.5 (0.9-13.9)	0.075	3.1 (0.8–12.0)	0.100
45–54	477	9.4	(6.3–13.8)	*7.7 (2.5–24.0)*	*0.000*	*5.4 (1.7–17.1)*	*0.004*
55–64	418	11.4	(8.2–15.7)	*9.3 (2.8–31.2)*	*0.000*	*6.9 (2.1–23.1)*	*0.002*
65+	685	11.0	(8.1–14.8)	*9.0 (2.6–31.1)*	*0.001*	d	d
CASMIN educational classification							
Low	806	8.7	(6.4–11.8)	1.1 (0.7–1.7)	0.768	−	−
Medium	1069	5.7	(4.3–7.5)	0.7 (0.4-1.1)	0.109	−	−
High	538	8.2	(5.8–11.4)	Ref	Ref	−	−
Income							
Low	656	6.1	(4.3–8.6)	Ref	Ref	−	−
Medium	1414	7.2	(5.7–9.2)	1.2 (0.8–1.8)	0.374	−	−
High	358	9.5	(6.1–14.4)	1.6 (0.9-2.8)	0.127	−	−
Employment situation							
Worker	844	7.9	(5.8–10.7)	1.0 (0.6–1.5)	0.890	−	−
Employee	773	8.1	(6.0–10.9)	Ref	Ref	−	−
Civil servant	183	7.0	(3.7–12.8)	0.9 (0.4–1.7)	0.659	−	−
Free-lance/self-reliant	293	8.5	(5.6–12.5)	1.0 (0.6–1.8)	0.880	−	−
Family worker	4	−	−	−	−	−	−
Other	64	0.9	(0.1–6.5)	*0.1 (0.1–0.1)*	*0.031*	−	−
Smoking, currently							
Daily	524	6.3	(4.3–9.2)	0.9 (0.6–1.5)	0.761	−	−
Occasionally	151	3.8	(1.7–8.2)	0.6 (0.2-1.3)	0.182	−	−
No, not anymore	899	9.0	(6.8–11.7)	1.3 (1.0-1.8)	0.089	−	−
Never smoked	843	6.8	(5.1–9.0)	Ref	Ref	−	−
Number of other children in household during childhood							
0	257	5.5	(3.2–9.3)	Ref	Ref	−	−
1–2	999	5.8	(4.3–7.9)	1.1 (0.6–1.9)	0.839	−	−
> 2	1172	8.9	(7.2–11.1)	1.6 (0.9-2.8)	0.087	−	−
Visit of nursery during childhood^d^							
No	561	10.8	(7.9–14.5)	*2.4 (1.7–3.5)*	*0.000*	*1.5 (1.0–2.2)*	*0.036*
Yes	1153	4.4	(3.4–5.8)	Ref	Ref	Ref	Ref
HSV1 serostatus							
HSV1 seronegative	548	6.5	(4.6–9.0)	Ref	Ref	−	−
HSV1 seropositive	1880	7.4	(6.0–9.1)	1.1 (0.8–1.7)	0.494	−	−
Degree of urbanization							
Rural	435	6.5	(3.2–12.6)	Ref	Ref	−	−
Provincial	620	6.1	(3.9–9.4)	0.9 (0.4–2.1)	0.875	−	−
Urban	712	7.7	(5.7–10.3)	1.2 (0.6–2.5)	0.660	−	−
Metropolitan	661	7.9	(6.2–10.2)	1.2 (0.6–2.5)	0.582	−	−
Region of residence							
West Germany	1675	6.6	(5.2–8.4)	Ref	Ref	−	−
East Germany (including Berlin)	753	9.2	(7.0–11.9)	1.4 (1.0-2.0)	0.070	−	−
German mother tongue							
Yes	151	7.4	(6.1–8.9)	Ref	Ref	−	−
No	2256	5.7	(3.2–9.9)	0.8 (0.4–1.4)	0.394	−	−
Number of sexual partners in last 12 months							
None	486	8.3	(5.5–12.3)	1.1 (0.7–1.8)	0.594	−	−
1	1613	7.3	(5.8–9.1)	Ref	Ref	−	−
2–3	155	5.3	(2.6–10.7)	0.7 (0.3–1.5)	0.395	−	−
> 3	73	6.9	(2.8–16.2)	0.9 (0.4–2.4)	0.905	−	−
Use of condoms							
Generally	289	2.7	(1.5–4.9)	Ref	Ref	Ref	Ref
Occasionally	314	6.8	(4.2–10.8)	*2.5 (1.1–5.7)*	*0.024*	*2.3 (1.0–5.1)*	*0.050*
No	1454	8.0	(6.4–9.9)	*3.0 (1.6–5.5)*	*0.001*	1.7 (0.9–3.2)	0.105
N/a	275	9.9	(5.7–16.5)	*3.7 (1.6–8.6)*	*0.003*	1.6 (0.6–4.7)	0.345

In italics: PR is statistically significant (*p* < 0.05)

^a^We used survey weights to account for deviations of the survey sample from the sampling parameters (i.e. age, sex, region, urban/rural region, community size, citizenship and education)

^b^If a *p*-value was <0.2 in the univariable analysis, we included that variable in a stepwise forward variable selection to find a suitable multivariable model

^c^Adjusted prevalence rates (PR) of variables which stayed in the final model (*p*-value ≤0.05) are reported

^d^Men older than 65 years of age were not asked whether they visited a nursery

### Changes in HSV1 seroprevalence from 1997–1999 to 2008–2011

**Fig. 3:**
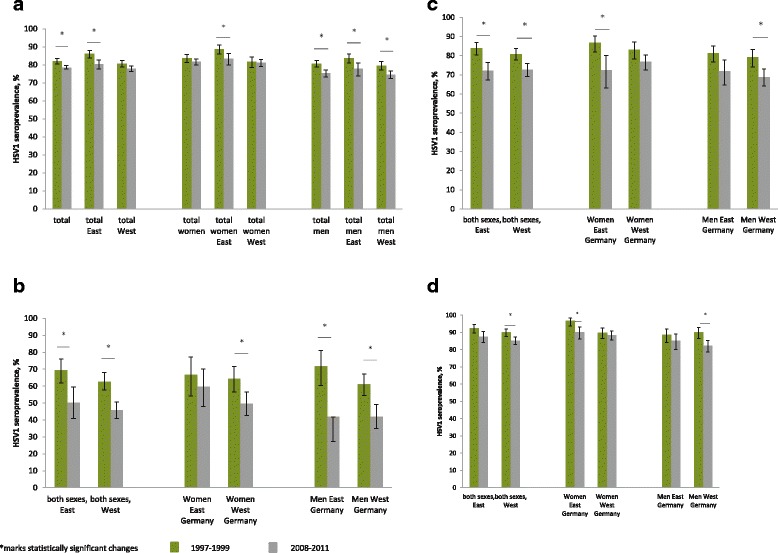
Change of HSV1 seroprevalence in Germany between 1997–1999 and 2008–2011, according to sex and region. **a** in adults aged 18–65 years, **b** in adults aged 18–24 years, **c** in adults aged 25–44 years, **d** in adults aged 45–64 years

Since the GNHIES survey in 1997–1999, the overall seropositivity for HSV1 in Germany has declined statistically significantly (Fig. 3a–d). Whereas the total adult HSV1 seroprevalence was 82.1% (95%CI 80.6–83.6) in the GNHIES, it was 78.4% (95%CI 77.8–79.7) in the DEGS. The decline was significant in men (1997–1999: 80.7%, 95%CI 78.7–82.5; 2008–2011: 75.3%, 95%CI 73.3–77.1) but not in women (1997–1999: 83.7%, 95%CI 81.4–85.8; 2008–2011: 81.6%, 95%CI 79.8–83.3). When stratified by region of residence, the decline was significant in both men (1997–1999: 83.6, 95%CI 81.0–86.0; 2008–2011: 77.7%, 95%CI 73.8–81.1) and women (1997–1999: 88.7%, 95%CI 86.0–91.0; 2008–2011: 83.3%, 95%CI 79.8–86.3) in East Germany, but only for men in West Germany (1997–1999: 79.5%, 95%CI 77.0–81.8; 2008–2011: 74.6%, 95%CI 72.3–76.7) (Fig. 3a).

In the group aged 18–24 years, HSV1 seropositivity declined from 64.0% (95%CI 59.1–68.5) in 1997–1999 to 46.7% (95%CI 42.4–51.1) in 2008–2011 (Fig. 3b). This effect was significant for both sexes, but more pronounced in men (1997–1999; 63.1%, 95%CI 57.4–68.5; 2008–2011: 41.9%, 95%CI 35.5–48.6) than in women (1997–1999: 64.8%, 95%CI 58.1–71.0; 2008–2011: 51.6%, 95%CI 45.6–57.6) in this age group. When stratified by region of residence, HSV1 seroprevalence declined significantly from 71.9% (95%CI 60.4–81.0) to 41.8% (95%CI 27.2–58.0) among 18–24-year-old men in East Germany, from 61.1% (95%CI 54.6–67.2) to 41.9% (95%CI 35.0–49.2) among 18–24-year-old men in West Germany, and from 64.3% (95%CI 56.5–71.5) to 49.7% (95%CI 42.8–56.6) among 18–24-year-old women in West Germany. Among 18–24-year-old women in East Germany, the decline in HSV1 seropositivity from 66.8% (95%CI 54.3–77.3) to 59.6% (95%CI 48.0–70.2) was not significant.

In 1997–1999, women aged 25–44 years in East Germany were more likely to be HSV1 seropositive (86.7%, 95%CI 81.9–90.3) than their counterparts in West Germany (83.1%, 95%CI 78.2–87.1), but the situation was reversed in 2008–2011 (East Germany: 72.5%, 95%CI 63.2–80.2; West Germany: 76.7%, 95%CI 72.6–80.4) (Fig. 3c). HSV1 Seropositivity in men aged 25–44 years decreased in East Germany (81.3%, 95%CI 76.8–85.1 to 71.8%, 95%CI 64.8–77.8) and West Germany (79.1%, 95%CI 74.2–83.2 to 68.8%, 95%CI 64.1–73.1).

### Changes in HSV2 seroprevalence from 1997–1999 to 2008–2011

**Fig. 4:**
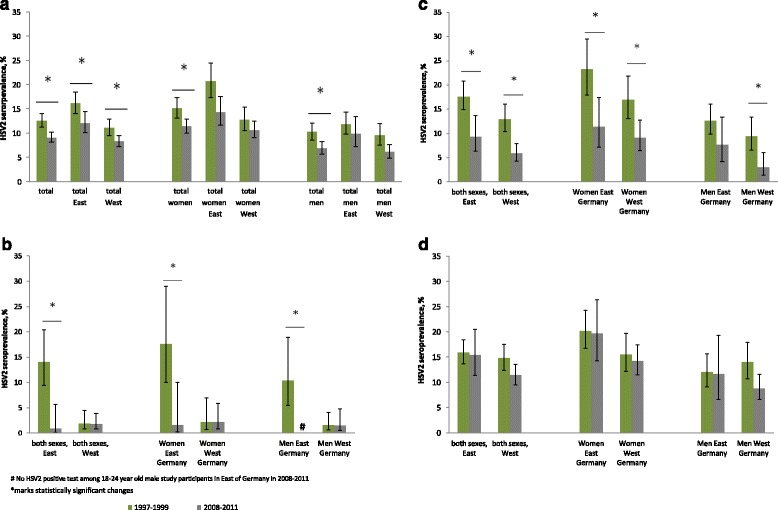
Change of HSV2 seroprevalence in Germany between 1997–1999 and 2008–2011, according to sex and region. **a** in adults aged 18–65 years, **b** in adults aged 18–24 years, **c** in adults aged 25–44 years, **d** in adults aged 45–64 years

The overall HSV2 seropositivity in Germany decreased significantly from 13.3% (95%CI 11.9–14.9) to 9.6% (95%CI 8.6–10.8) in these years (Fig. 4a–d). The decline was significant for both women (1997–1999: 16.0%, 95%CI 13.9–18.4; 2008–2011: 12.1%, 95%CI 10.6–13.7) and men (1997–1999: 10.9%, 95%CI 9.2–12.8; 2008–2011: 7.3%, 95%CI 6.0–8.8). When stratified by region of residence, the decline was significant in both East Germany (1997–1999: 17.1%, 95%CI 14.9–19.6; 2008–2011: 12.8%, 95%CI 10.7–15.3) and West Germany (1997–1999: 11.8%, 95%CI 10.1–13.7; 2008–2011: 8.8%, 95%CI 7.6–10.1).

In the study participants aged 18–24 years at the time of the surveys, there was a decline in HSV2 seroprevalence from 4.2% (95%CI 2.6–6.5) to 1.6% (95%CI 0.8–3.3). No change in HSV2 seroprevalence was observed in this age group when the study participants lived in West Germany (men: from 1.6% [95%CI 0.6–4.1] to 1.5% [95%CI 0.5–4.8]; women: 2.2% [95%CI 0.7–6.9] to 2.2% [95%CI 0.8–6.0]), but in East Germany, HSV2 seropositivity for men declined from 10.4% (95%CI 5.5–18.9) to 0% and for women from 17.6% (95%CI 10.0–29.0) to 1.6% (95%CI 0.2–10.0) (Fig. 4b).

In the age group 25–44 years, HSV2 seropositivity dropped from 14.4% (95%CI 12.4–16.7) to 6.5% (95%CI 5.1–8.4), and when stratified by sex and region of residence, this reduction was significant for women in East Germany (from 23.2% [95%CI 17.9–29.5] to 11.4% [95%CI 7.2–17.4]), women in West Germany (from 16.9% [95%CI 13.2–21.8] to 9.1% [95%CI 6.4–12.7]), and men in West Germany (from 9.4% [95%CI 6.5–13.4] to 3.0% [95%CI 1.4–6.0]), but not for men in East Germany (from 12.6% [95%CI 9.8–16.0] to 7.6% [95%CI 4.2–13.4]) (Fig. 4c).

## Discussion

Based on two population-based surveys, we estimated that the HSV1 seroprevalence in adult Germans decreased from 82.1% in 1997–1999 to 78.4% in 2008–2011. Declines in HSV1 seroprevalence have been seen in many countries worldwide, including the USA [25], England and Wales [26], Israel [27], and the Netherlands [28]. Fewer siblings in industrialized countries, less-crowded institutions, improvements in living conditions, and better hygiene have been suggested to explain for these declines [25]. We assume that many factors contributed to the decrease of HSV1 seroprevalence in Germany. The increasing number of single households (35% in 1998, 40% in 2010) and the decreasing number of households with three or more generations in Germany (by 41% between 1995 and 2015) [29] could have generally led to a reduced chance to transmit the virus. Hypotheses concerning the sexual transmission routes include the increased use of condoms in young adults (women from 68% to 75%, men from 55% to 76% between 1998 and 2009) and a higher coverage of sexual health education at schools [20].

A reduced seroprevalence of HSV1 has also been linked to more symptomatic HSV2 infections and more cases of genital HSV1, both of which pose a threat to neonates whose mothers acquire the infection in the third trimester of pregnancy [1, 30]. Therefore, the decline in HSV1 seroprevalence, especially its decline in young adults, is of concern.

We estimated a decline in the seroprevalence of HSV2 in adult Germans from 13.3% in 1997–1999 to 9.6% in 2008–2011. A reduction in HSV2 seroprevalence between 1984 and 2002 has also been reported in young adults in Israel [28, 27]. Although reductions in HSV2 seroprevalence were observed in 20–24-year-old men and 15–19-year-old women in the Netherlands between 1995– 1996 and 2006–2007 (14), the overall seroprevalence of HSV2 in the Netherlands remained stable during that period (14). In the USA, HSV2 seroprevalence did not change significantly between 1999 and 2010 [25]. The reduced seroprevalence of HSV2 in Germany leave more people susceptible to genital HSV infections which, again, especially combined with the reduced HSV1 seroprevalence, is a threat to neonates whose mothers acquire the infection in the third trimester of pregnancy [1, 30].

The seroprevalence of both HSV1 and HSV2 was higher in women than in men in Germany. The greater biological susceptibility of women to infection by genital transmission is well known [4]. Because the genital disease is more likely to be asymptomatic in men [31], men are likely to be underdiagnosed and to continue to engage in sexual activities, increasing male-to-female transmission. We found increasing seroprevalence of both HSV1 and HSV2 with increasing age, which is consistent with other studies and correlates with cumulative exposure [17, 32].

Less-educated women and men were more likely to be HSV1 seropositive in our study. It is well known that education plays an important role in the prevention of infections. A lower level of education is probably associated with a lower parental income and cheaper housing during childhood, although we did not examine these relationships in our survey. HSV1 acquisition is also known to be linked to socioeconomic status and a crowded living environment [33]. Interestingly, compared with never-smokers, women and men in our survey who currently smoked were more likely to be HSV1 seropositive, which may be attributable to the usually lower socioeconomic status of smokers [34] or reflect the lowered (local) immune response caused by cigarette smoking. Not speaking German as the first language was also associated with HSV1 seropositivity in both sexes. However, the DEGS did not adequately represent non-Germans and the number of study participants was low in the subset of non-German speakers (Tables 1 and 2).

Smoking was associated with HSV2 seropositivity in women but not in men when the data were adjusted for age. Interestingly, women who had had one or more abortion were 1.5 times more likely to be HSV2 seropositive. Genital herpes is not an indication for medically induced abortion [35]. We strongly encourage further studies to analyze this finding [36, 37]. In men, the multivariable analysis revealed a positive association between HSV2 IgG and not having attended a nursery school during childhood, which is consistent with the theory that low exposure to HSV1 during childhood increases the risk of genital HSV2 (or HSV1) acquisition later in life [38]. However, we found no correlation between HSV1 serostatus and HSV2 serostatus in the multivariable analysis. Men who only occasionally used condoms were more likely to be HSV2 seropositive than men who consistently used condoms. However, men who did not use condoms at all were not more likely to be HSV2 seropositive than men who consistently used condoms.

In a previous survey based on the GNHIES data from 1997 to 1999, HSV2 seroprevalence was higher in women living in East Germany than in women living in West Germany [18]. Although we still observed this trend in the older age groups in the survey performed in 2008–2011, there was a remarkable decline in HSV2 seroprevalence between 1997–1999 and 2008–2011 in both women and men aged 18–24 years residing in East Germany. In this age group, HSV2 seroprevalence no longer differed between East and West Germany, regardless of sex. Because young people aged 18–24 years in 2008–2011 were born and raised around the time of the reunification of Germany in 1990, this finding may be partly attributable to the change in attitude to the prevention of sexually transmitted diseases and the change in sexual behavior in East Germany since 1990. In the former GDR, hormonal contraception was predominant and available free of charge from the age of 14 years [19]. After reunification, the pill only remained free for women under the age of 20 years and condoms became more easily available and more popular in East Germany. Moreover, in East Germany before 1990, adolescents tended to establish a family to gain the right to rent an apartment, whereas remaining single became popular after reunification because a flexible and independent individual has a greater chance of employment in the free market economy. Reunification also brought changes in the abortion law, the perception of homosexuality, divorce, prostitution, and pornography. Altogether, these changes probably resulted in a higher awareness of HIV, greater condom use, and consequently fewer HSV2 infections in the 18–24-year-olds residing in East Germany.

There were a number of limitations to our study. Both surveys collected data with research questions that focused on non-communicable diseases. Only very few questions about sexual behavior were included. Furthermore, the participants’ HIV status or affiliations to a group with an increased risk of STI were not known. The study participants were not asked about the site of their infection. Because seropositivity does not distinguish between different routes of transmission, we were unable to draw any conclusions about the ratio of genitally to non-genitally acquired HSV1 and HSV2 infections. Moreover, although we measured seroprevalence, we did not investigate the burden of the disease, such as the number of clinical episodes, or the number of infected neonates and the grade of severity. A number of items in the questionnaire only covered the past year of the study participant’s life and it is possible that their behavior had been different before that period. Making assumptions about the determinants of a lifelong infection is difficult in a cross-sectional study. Lastly, non-German citizens were underrepresented in both surveys and we were unable to investigate the differences in HSV1/2 seropositivity between persons with German or non-German nationality.

## Conclusions

The estimated seroprevalence of both HSV1 and HSV2 imposes large clinical and psychosocial burdens on Germany. Therefore, the declines in HSV1 and HSV2 seroprevalence between the late 1990s and 2008–2011 benefit the nation. However, they also have a negative consequence, because they increase the susceptibility of sexually active people to genital HSV infections, including pregnant women. Therefore, practitioners should be aware of HSV infection as a differential diagnosis for genital ulcers, and should improve the counseling of affected patients and optimize the diagnosis, treatment, and prevention. This includes asking women at their first antenatal visit if they or their partner have had herpes, offering an explanation of possible preventive strategies, and counseling parents to avoid direct contact between herpetic lesions and the neonate [35, 39]. We recommend educational interventions to raise awareness of the sexual transmission route of the infection, of its possible consequences, and of its prevention. Interventions should especially target pregnant women and their partners, as well as people at risk of HIV and their partners.

It is important to continue to measure the seroprevalence of HSV1 and HSV2 in Germany over time to monitor trends, and it is essential to expand the data collected in future surveys to include a comprehensive set of questions regarding sexual behavior and HIV status. This will allow the influence of HSV infections on the HIV epidemic (and vice versa) in Germany to be assessed. We also encourage studies that explore the proportions of HSV1 and HSV2 infections among genital infections in Germany and to measure and monitor the number of neonatal HSV infections.

## Abbreviations

CASMIN: Comparative Analysis of Social Mobility in Industrial Nations
CI: Confidence Interval
CPR: Crude prevalence ratio
DEGS: German Health Interview and Examination Survey for Adults
ELISA: Enzyme-linked immunosorbent assay
FRG: Federal Republic of Germany
GDR: German Democratic Republic
GNHIES: German National Health Interview and Examination Survey
HIV: *Human immunodeficiency virus*
HSV: Herpes simplex virus
OD: Optical density
PR: Prevalence ratio
STI: Sexually transmitted infection
